# Immunological Features of Paranasal Sinus Mucosa in Patients with Graves’ Orbitopathy

**DOI:** 10.3389/fendo.2020.621321

**Published:** 2021-02-10

**Authors:** Yi Lu, Yu Wu, Yazhuo Huang, Sijie Fang, Yinwei Li, Jing Sun, Huifang Zhou

**Affiliations:** ^1^ Department of Ophthalmology, Shanghai Ninth People's Hospital, Shanghai JiaoTong University School of Medicine, Shanghai, China; ^2^ Shanghai Key Laboratory of Orbital Diseases and Ocular Oncology, Shanghai Ninth People's Hospital, Shanghai, China

**Keywords:** immune, T cells, paranasal sinus, mucosa, Graves’ orbitopathy

## Abstract

**Background:**

Previous studies showed that patients with Graves’ orbitopathy (GO) had concomitant mucosal abnormality within the paranasal sinuses. It remains unknown whether the immunological reactions in sinus mucosa affect the orbit inflammation in GO.

**Methods:**

Patients with GO underwent sinus computed tomography (CT) scans for sinus mucosal disease by two independent reviewers using the Lund-MacKay systems. Ethmoid mucosal samples were collected during orbital decompression surgeries for patients with GO and correction surgeries for patients with old orbital fractures as controls. Histological analysis and immunofluorescence were performed in all sinus mucosa tissues. Flow cytometry analysis was used to examine the immunological features of sinus mucosa in both GO and control groups.

**Results:**

Immunohistochemistry showed that the paranasal sinus mucosa of patients with GO grew swelling, with goblet cell and small vessel proliferation, endothelial cell swelling, and inflammatory cell infiltration. The number of T helper (Th)1, Th17, and gamma-delta T cells in nasal sinus mucosa of patients with GO increased significantly compared with those from controls. Further, the proportion of Th1 cells was significantly correlated with clinical activity score. In addition, there was a decreased number of regulatory T cells in patients with GO. The number of Th2 cells showed no significant difference between the two groups. Finally, the proportion of interleukin-22-producing cell subsets in gamma-delta T cells of patients with GO was significantly increased compared with those from controls.

**Conclusions:**

Our observations illustrated a potential pathogenic role of mucosal-infiltrating T cells, which may have the possibility to aggravate inflammatory responses in GO.

## Introduction

Graves’ orbitopathy (GO) is an autoimmune disorder ranking first in orbital diseases. GO is also the most common extrathyroidal manifestation of Graves’ disease (GD) (1). Orbital tissue remodeling is a major pathological feature of GO, which includes extraocular muscle swelling, adipogenesis, fibrosis, and extracellular matrix deposition such as glycosaminoglycan (2). It is generally accepted that the bony orbit confines the orbital soft tissues on all sides except anteriorly. Intriguingly, the two most frequently enlarged extraocular muscles are the inferior and medial rectus muscles, which are next to ethmoid and maxillary sinus, respectively. Both muscles overlie the thinnest bones of the orbit (3). Gouveris et al. reported that patients with GD had concomitant mucosal thickening within the paranasal sinuses. Patients with GO who underwent orbital decompression surgeries exhibited a higher prevalence (29.4%) of sinus mucosal thickening on CT scan. Histological exams showed chronic rhinosinusitis in 45.5% of GO patients (4). These findings may be explained by circulating autoantibodies targeting autoantigens, namely thyrotropin receptor and insulin-like growth factor-1 receptor, associated with GD and found to be expressed in sinus mucosa (5).

Until today, the pathogenesis of GO is not fully understand. Many studies have indicated that T cells are critical in GO autoimmune responses. They have confirmed that at least three T helper (Th) cell groups are involved in the development of GO, which are Th1 cells, Th2 cells, and Th17 cells. Early studies mainly focused on Th1/Th2 subsets and their related cytokines in the inflammation of GO. It is recognized that Th1 cells play a significant role in inducing cytokine release in early active GO, and the chronic fibrotic phase of GO is dominated by Th2-type immune responses (6–9). Furthermore, Th17 cells have been proved to be an important proinflammatory and profibrotic cell subsets. In GO patients, the level of interleukin (IL)-17A and the number of IL-17A-producing T cells in the peripheral blood were higher than those of healthy controls. IL-17A was shown to promote inflammation and fibrosis of orbital fibroblasts (OF) derived from GO patients (10–12). Moreover, Th17 cells could stimulate the expression of proinflammatory cytokines such as IL-6, IL-8, monocyte chemoattractant protein-1, tumor necrosis factor-α, etc., and costimulatory molecules to regulate the balance of fibrosis and adipogenesis in different OF subsets (13). In this current study, we raise a hypothesis that there is a relationship between nasal sinuses inflammation and GO pathology. We sought to investigate why there is a higher prevalence of sinus mucosa disorder in GO patients and to unravel the potential immunological features of paranasal sinus mucosa in GO.

## Materials and Methods

### Subjects and Samples

A collection of 58 ethmoid sinus mucosa tissues (38 samples of patients with GO undergoing orbital decompression surgery and 20 samples of patients with old orbital fracture undergoing orbital repair surgery) were included in the study. Informed consent was provided by all participants. Use of these samples was approved by the Ethical Committee of Shanghai Ninth People’s Hospital, Shanghai JiaoTong University School of Medicine. All 38 GO patients were in inactive stage. Steroid or immunosuppressive treatment was discontinued for at least 3 months before surgery, and all patients were clinically euthyroid at the time of surgery. Exclusion criteria included age less than 18 years old, and patients with history of sinus surgery. Patients with rheumatic or hemolymph diseases were also excluded. Based on the findings of pre-operative computer tomography (CT) scans, scores based on the Lund-Mackay staging systems (**Table 1**) were calculated for each patient and control subject.

**Table 1:** Lund-Mackay Scoring System.

Anatomic Location	Scoring Criteria*
Maxillary sinuses	0: No abnormality
	1: Partial opacification
	2: Total opacification
Anterior ethmoid sinuses	0: No abnormality
	1: Partial opacification
	2: Total opacification
Posterior ethmoid sinuses	0: No abnormality
	1: Partial opacification
	2: Total opacification
Sphenoid sinuses	0: No abnormality
	1: Partial opacification
	2: Total opacification
Frontal sinuses	0: No abnormality
	1: Partial opacification
	2: Total opacification
Ostiomeatal complex (OMC)	0: Not occluded
	2: Occluded

*Total score can range from 0 to 24.

### Histology and Immunofluorescence

For histological analysis, nasal sinus mucosa tissues were fixed in 4% formalin and paraffin-embedded. The specimens were cut in 4-µm-thick sections before staining with hematoxylin and eosin (HE). The sections were then examined with a light microscope. The expression of CD4 and CD8 was determined by immunofluorescence analysis. Cryostat sections (4 μm thick) were fixed with 4% paraformaldehyde fixative at room temperature for 20 min and washed in PBS for three times. The sections were then incubated with a blocking solution (normal goat serum) at room temperature for another 20 min. Next, they were incubated with rabbit anti-CD4 and mouse anti-CD8 (Abcam, USA) primary antibodies overnight at 4°C. After being washed in PBS for three times, the sections were incubated with secondary antibodies for 1 h at room temperature. Finally, coverslip-mounted slides were observed and photographed under a fluorescence microscope.

### Flow Cytometry Analysis

The ethmoid sinus mucosa tissues were digested with 5% Type IV collagenase into single cell suspensions, and were stimulated with phorbol 12-myristate 13-acetate (50ng/ml; Sigma-Aldrich, USA) and ionomycin (1 μg/ml; Sigma-Aldrich, USA) in the presence of Golgi Plug (1 μl/ml; BD Biosciences, USA) at 37°C for 6 h before flow cytometry. To analyze T cell subsets in sinus mucosa, cells were incubated with APC-Cy7-Fixable Viability Dye (eBioscience, USA) before staining for surface markers (FITC-anti-CD3, AF700-anti-CD8, BV650-anti-γδTCR) (All from BD Biosciences, USA). Then, cells were treated with Fixation/Permeabilization reagents (eBioscience, USA) and stained with intracellular markers (BV711-anti-IFN-γ, BV421-anti-IL-13; BD Biosciences, USA; PE-anti-IL-17A, APC-anti-FOXP3, PE-Cy7-anti-IL-22; eBioscience, USA). A BD LSRFortessa X-20 was applied for sample sorting. The data were analyzed after adjusting the fluorescence compensation with FlowJoV10.

### Statistical Analysis

All values are expressed as the mean ± standard deviation (SD). Statistical analyses were performed by one-way ANOVA, analysis of Spearman’s correlation and t-test followed by the appropriate tests with the statistical analysis program GraphPad Prism 6.0 (GraphPad Prism Software, USA) and SPSS 25 (IBM SPSS Software, USA). A *P* value less than 0.05 was considered to indicate a statistically significant difference.

## Results

### Clinical Characteristic and Radiographic Findings of GO Patients

**Figure 1:**
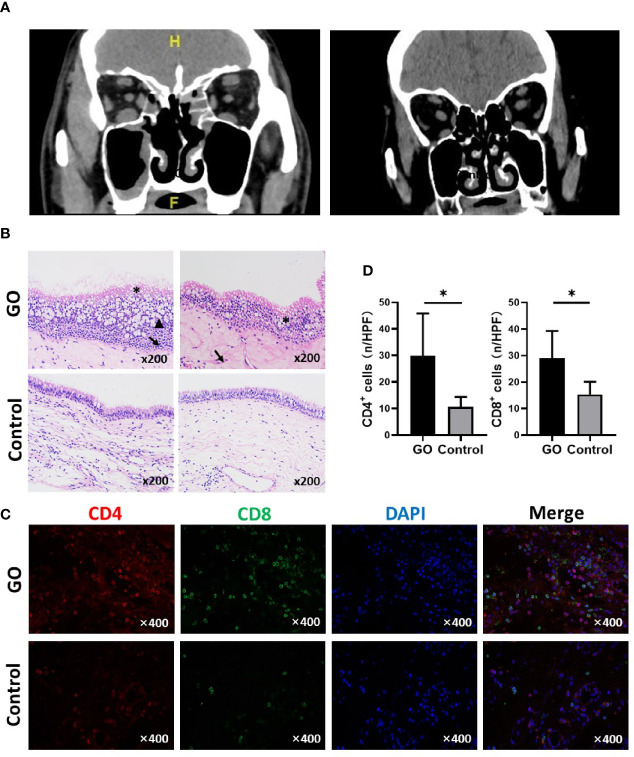
Orbital computed tomography (CT) images and nasal sinus mucosa histological presentation of Graves’ orbitopathy (GO) patients and controls. **(A)** Left: A GO patient shows mucosal thickening of maxillary sinus and ethmoid sinus on both sides; Right: Normal control with right orbital fracture. **(B)** Typical changes include mucosal swelling and epithelial cells proliferation. *Disorder of ciliary columnar epithelial; ↑epithelial cell proliferation; ^▲^ Mucus over-secretion. (HE staining, ×200). **(C)** Immunofluorescence staining results of CD4 and CD8 positive cells in GO and control mucosa. **(D)** Statistical analyses of CD4 and CD8 positive cells, the mucosa of GO has a significant increase compared with control. Significance (*P*) values after t-test was represented by *P < 0.05.

Clinical characteristics of patients and controls were described in **Table 2**. As expected, the GO group had more proptosis reflected in higher exophthalmometry readings than controls (23.37 ± 2.44 vs. 15.24 ± 2.73, *P* < 0.0001) mm. CT scans demonstrated a higher score in patients with GO compared with controls for Lund-MacKay system (4.00 ± 1.94 vs. 1.67 ± 0.58, *P* < 0.0001). Among GO patients, the most common location for mucosal thickening was in the ethmoid sinuses (69.2%), followed by the maxillary (53.8%), OMC (46.2%), and sphenoid sinuses (30.8%). The representative CT scanning was showed in **Figure 1A**. When comparing the extent of sinus mucosal thickening to the degree of orbital proptosis as measured by exophthalmometry, higher Lund-MacKay scores did not correlate with greater orbital proptosis (r=-0.1386, *P* =0.582).

**Table 2:** Clinical Characteristic and Radiographic Finding of GO and normal control.

	GO	Control	*P* value
Number, n	38	20	
Male/Female, n/n	16/22	11/9	0.41
Age(years), mean **±** SD	44.80 ± 11.64	40.91 ± 12.21	0.38
Smoker, n (%)	13 (34.2%)	11 (55.5%)	0.16
Proptosis(mm), mean **±** SD	23.65 ± 2.31	15.24 ± 2.73	<0.0001
Course of disease (months), mean **±** SD	23.84 ± 20.62	/	
Sinus mucosal thickening, n (%)	15 (39.5%)	3 (15%)	0.08
Lund-Mackay Score, mean **±** SD	3.84 ± 1.83	1.67 ± 0.58	<0.0001
Involved nasal sinuses, n (%)			
Ethmoid	11 (73.3%)	1 (33.3%)	0.25
Maxillary	9 (60.0%)	3 (100%)	0.51
OMC	6(40.0%)	0	0.51
Frontal	0	0	1
Sphenoid	4 (26.7%)	1 (33.3%)	1

Mucosal thickening defined as Lund-MacKay score≥1.

GO, Graves’ orbitopathy; CT, computed tomography; SD, standard deviation; OMC, osteomeatal complex.

### Histological Changes of Paranasal Sinus Mucosa in GO Patients

Histological changes of the sinus mucosa were found in 21 out of the 38 GO patients (60%). The mucosa showed thickening in GO patients. Compared with normal nasal sinus, typical histological changes include mucosal swelling and epithelial cell proliferation. Some cases show goblet cell proliferation and mucus over-secretion; chronic inflammatory cell infiltration and small vessel proliferation (**Figure 1B**). CD4^+^ and CD8^+^ T cells infiltration can be found in GO patients’ mucosa, which was detected by immunofluorescence staining (**Figure 1C**). There was a significant increase of CD4^+^ (29.78 ± 15.16 vs. 10.60 ± 3.78, *P*=0.024)/HPF and CD8^+^ T cells (29.00 ± 10.33 vs. 15.33 ± 4.76, *P*=0.013)/HPF in GO mucosa compared with controls (**Figure 1D**).

### Immunological Characteristics of Paranasal Sinuses Mucosa in GO

**Figure 2:**
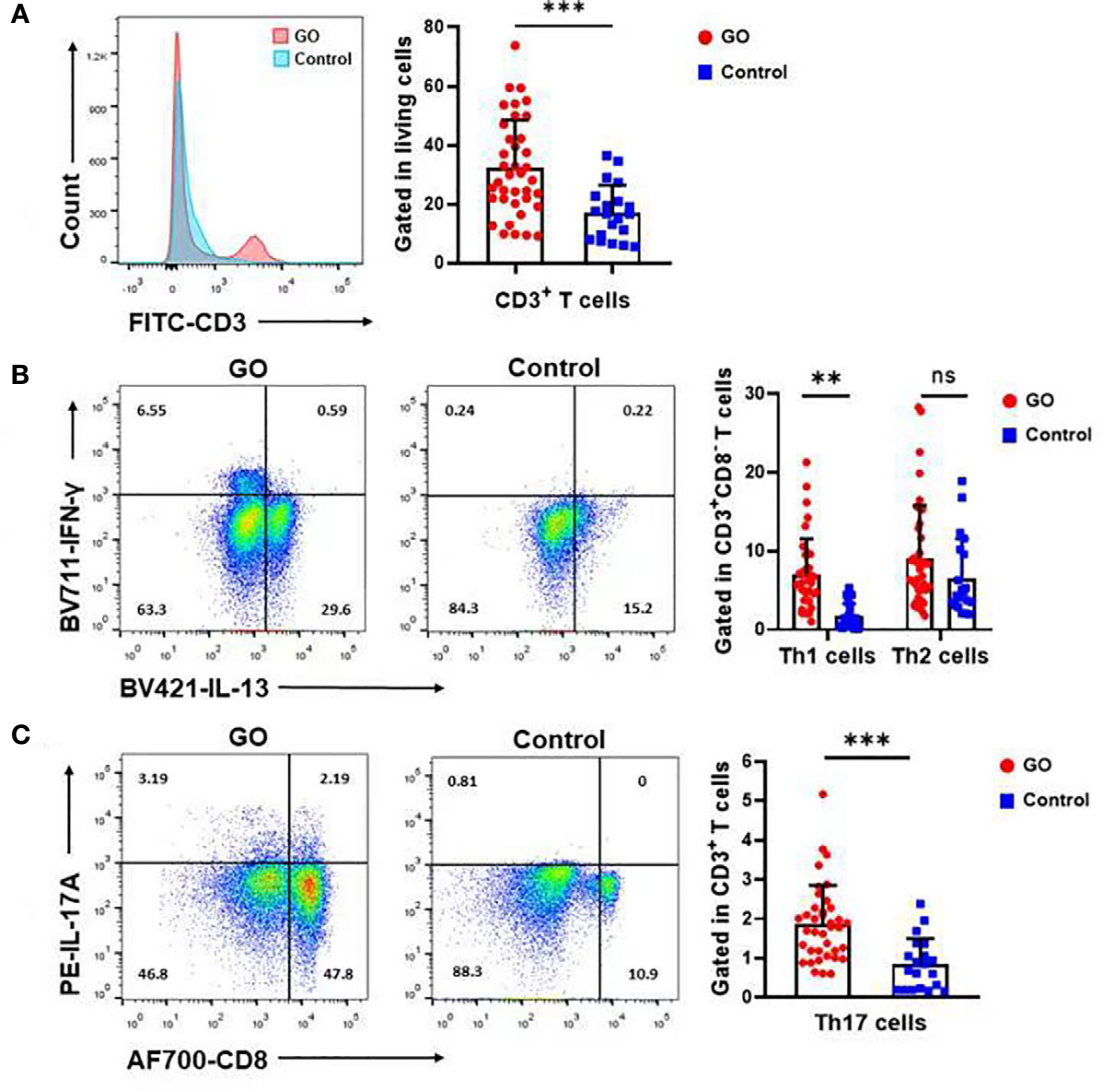
Th1, Th17 but not Th2 cells varied in Graves’ orbitopathy (GO) patients compared with controls. **(A)** The percentage (%) of CD3^+^ cells to total living cells was higher in GO patients compared with controls. **(B)** Flow cytometry showed that Th1 cells increased significantly in GO patients’ mucosa, while Th2 cells showed no significant difference between GO patients and controls. Significance (*P*) values after t-test was represented by **P < 0.01, ***P < 0.001. **(C)** Flow cytometry showed that Th17 cells increased significantly in GO patients’ mucosa. Significance (*P*) values after t-test was represented by ***P < 0.001.

**Figure 3:**
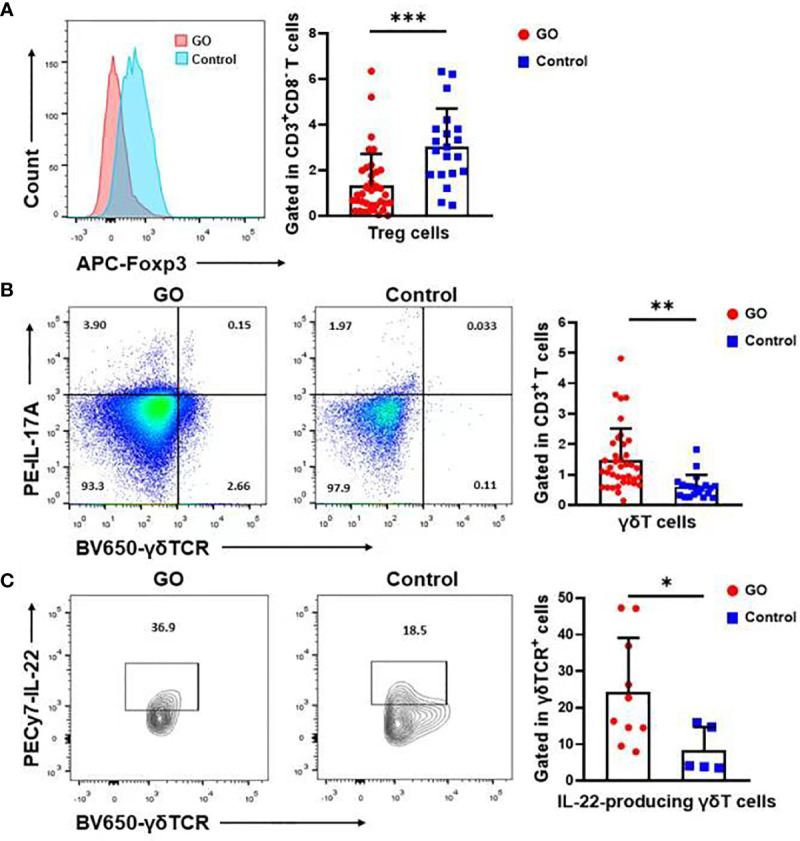
The significant change of Treg cells and γδT cells in the nasal sinus mucosa of Graves’ orbitopathy (GO) patients. **(A)** There was also a significant decrease in the percentage (%) of Treg cells in GO patients. Significance (*P*) values after t-test was represented by ***P < 0.001. **(B)** Flow cytometry showed that γδT cells increased significantly in GO patients’ mucosa. Significance (*P*) values after t-test was represented by **P < 0.01. **(C)** There was also a significant increase in the percentage (%) of IL-22 secreting γδT cells in GO patients. Significance (*P*) values after t-test was represented by *P < 0.05.

T cell populations present in the nasal mucosa from GO patients and normal controls were identified as CD4/CD8/γδ T cells by using flow cytometric analysis. The proportion (%) of CD3^+^ to total living cells (**Figure 2A**) was higher in GO patients compared with normal controls. (33.69 ± 15.84% vs.17.15 ± 9.04%, *P*<0.001). Th1 cells were defined as CD3^+^CD8^-^IFN-γ^+^ subsets and Th2 cells as CD3^+^CD8^-^IL-13^+^ subsets. Our data indicated that Th1 cells increased significantly in GO patients’ mucosa (7.19 ± 4.69% vs. 1.74 ± 1.67%, *P*<0.01), while Th2 cells showed no difference between GO patients and normal controls (9.37 ± 6.70% vs. 6.90 ± 5.10%, *P*=0.16) (**Figure 2B**). In patients with GO, the mucosa was also characterized by significantly increased Th17 cells (CD3^+^CD8^-^IL-17A^+^) (1.91 ± 0.99% vs. 0.84 ± 0.63%, *P*<0.001) (**Figure 2C**) and decreased Treg cells (CD3^+^CD8^-^FOXP3^+^) (1.38 ± 1.39% vs. 3.04 ± 1.62%, *P*<0.001) (**Figure 3A**), which suggested a similar Th-driven inflammation to orbital connective tissue. In addition to the changes of Th cells in the mucosa, we further investigated into the difference of γδ T cells between the two groups, which play an important role in mucosal immunity. Our results showed that γδT cells was significantly elevated in GO patients’ mucosa (1.47 ± 1.05% vs. 0.60 ± 0.38, *P*<0.01) (**Figure 3B**). Additionally, we examined IL-17A expression in those sinus mucosal γδT cells and found that there was no difference between the two groups. Intriguingly, we observed a significantly augmented amount of IL-22-secreting γδT cells in the mucosa of GO patients compare with controls (24.32 ± 14.76% vs. 8.42 ± 6.30%, *P*=0.04) (**Figure 3C**).

### The Relationship Between Th1, Th2, Th17 Cells in Nasal Sinus Mucosa and Clinical Characteristics of GO

**Figure 4:**
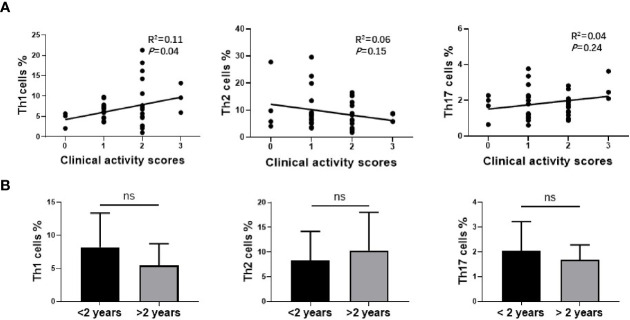
The relationship between Th1, Th2, Th17 cells in nasal sinus mucosa and clinical characteristics of Graves’ orbitopathy (GO). **(A)** The analysis of Spearman’s correlation between Th1, Th2, Th17 cells and CAS of GO patients. Th1 cells amount was correlated with CAS of GO patients significantly. **(B)** There was no significant difference between two groups of different disease course in Th1, Th2 or Th17 cells.

The proportion of different subtypes of T cells was further analyzed with GO clinical activity score (CAS). Our data indicated that the proportion of Th1 cells was significantly correlated with disease activity (R^2^ = 0.11, *P*=0.0474), while there was no correlation between the number of Th2 cells (R^2^ = 0.07, *P*=0,136) or Th17cells (R^2^ = 0.02, *P*=0.446) and CAS (**Figure 4A**). The study cohort was then divided into two groups according to the time course of GO. One group was longer than 2 years, and the other was less than 2 years. The subtypes of T cells were re-analyzed on the basis of disease course. Our data indicated that the average proportion of Th1 cells (8.23 ± 5.03% vs. 5.62 ± 3.40%) and Th17 cells (2.04 ± 1.16% vs. 1.74 ± 0.62%) declined with the extension of disease course, while Th2 cells (8.27 ± 5.77% vs. 11.11 ± 8.02%) slightly increased with GO duration. However, there was no significant difference in any of the T cell subtypes between these two groups (*P*=0.11, 0.40, 0.24) (**Figure 4B**), which may attribute to the limited number of cases.

## Discussion

Previous studies suggested that disorders of the paranasal sinus mucosa were associated with a number of systemic autoimmune diseases. Patients with GD were reported to have significantly greater paranasal sinus mucosal thickening on CT scan than normal controls. The presence of autoantigens TSHR and IGF-1R were also identified in paranasal sinus mucosa (5). These studies hold the opinion that autoantibodies circulating in the blood of patients with GD would be expected to bind to either TSHR or IGF-1R in the sinus mucosa, as occurs in the thyroids and orbital connective tissues, resulting in the localized mucosal edema and inflammatory cell infiltration.

T cell immunity plays an important role in the pathogenesis of GO (14). The frequency of Th1 cells and the Th1/Th2 ratio were positively correlated with the inflammatory activity score of GO (8). Wakelkamp et al. (15) found that active GO patients were characterized by Th1 type cytokines and there was no direct correlation between Th2 type cytokines and disease progression. Han *et al*. further demonstrated that IFN-γ secreted by Th1 cells and IL-4 secreted by Th2 cells can promote the production of hyaluronic acid by orbital fibroblasts. However, our research did find that there was no difference between GO and control groups in Th2 population. Some studies have revealed that Th1 cells may predominate in the orbit in early GO and Th2 cells might play a greater role in later stages of the disease (16, 17). Yet, others studies have indicated that the active phase is characterized by the presence of proinflammatory and Th1-derived cytokines, whereas other cytokines, including Th2-derived cytokines, do not seem to be linked to a specific stage of GO (18, 19). However, we found most of the studies outdated. Notably, these studies defined Th2 cells by means of establishing T cell clones or examining bulk RNA expression of cytokines within orbital connective tissues due to technical limitation at that time, which made their results not convincing enough. In order to explain the phenomenon, the characteristics of chronic sinusitis should be considered. Chronic rhinosinusitis (CRS) is a persistent inflammatory disease affecting paranasal sinuses. CRS is categorized into two distinct subgroups defined as CRS with nasal polyps (CRSwNP) and CRS without nasal polyps (CRSsNP) (20). Th1 cytokines are mostly found in CRSsNP and Th2 cytokines in CRSwNP. The sinus mucosal abnormality in GO patients isn’t usually accompanied by polyps, which might explain the Th1 but not Th2 dominance observed in our study cohort.

We and other groups reported that in GO patients the level of IL-17A and the number of IL-17A-producing T cells were higher than those of healthy controls in the periphery blood as well as in orbital connective tissues (10–12, 21, 22). These results indicate that the CD4^+^ T cells may contribute to the immunopathological process in GO. In this current study, we also found that there was a higher proportion of both Th1 cells and Th17 cells in the sinus mucosa of GO patients. Combined with previous studies, we suspect that these lymphocytes in nasal sinus may infiltrate into the orbit through the very thin bone walls and blood vessels.

Furthermore, we studied Treg cells in the sinus. Treg cells are a suppressive subset of CD4^+^ T subsets important for the regulation of immune responses in various autoimmune diseases. Treg cell dysfunction also contributes to the development of autoimmune thyroid disease (23–25). A study showed that patients with improved GO were more likely to have higher proportion of Treg cells than those with stable or deteriorated GO. Thus, the number of Treg cells in the peripheral blood of GO patients can be used as a predictor of clinical course (26). Kahaly et al. (27) demonstrated that the proportion of Treg cells in the peripheral blood leukocytes derived from GO patients increased after incubation with rabbit polyclonal anti-T lymphocyte globulin. Taken together, our findings reinforced the point of view that the decline of Treg cells might be responsible for the activation of inflammatory cells in GO.

Despite CD4^+^ and CD8^+^ T cells, γδT cells are a distinctive subset of T cells that were first recognized by Brenner in 1986. These cells primarily colonize in the mucosa and skin where they play an important role in immune regulation (28). In autoimmune disorders, γδT cells have immunoregulatory properties and secrete both IL-17A and IL-22 (29). A previous study showed that orbital-infiltrating T cells were primarily γδT cells, while the classic αβT helper cells were rare (30). Intriguingly, we previously confirmed an elevated subset of IL-17A-producing γδT cells in the circulation of GO patients compared with healthy controls (12). However, in this current study, we did not find the up-regulation of IL-17A-producing γδT cells, while IL-22-producing γδT cells were observed to be increased in GO sinus mucosa. IL-22 can affect the fibroblasts in the skins, intestines, and joints. Proliferation of synovial fibroblasts in rheumatoid arthritis patients as well as monocyte chemoattractant protein-1-dependent recruitment of monocytes to the sites of inflammation are driven by IL-22 (31). Furthermore, IL-22 can increase the production of anti-inflammatory factors such as IL-11 as well as inflammatory mediators such as IL-6 and CXCLs chemokines in human colonic myofibroblast cells (32, 33). These results indicate that the infiltration of γδT cells in sinus mucosa might be associated with the complicated and refined regulation of orbital inflammation in patients with GO.

**Figure 5:**
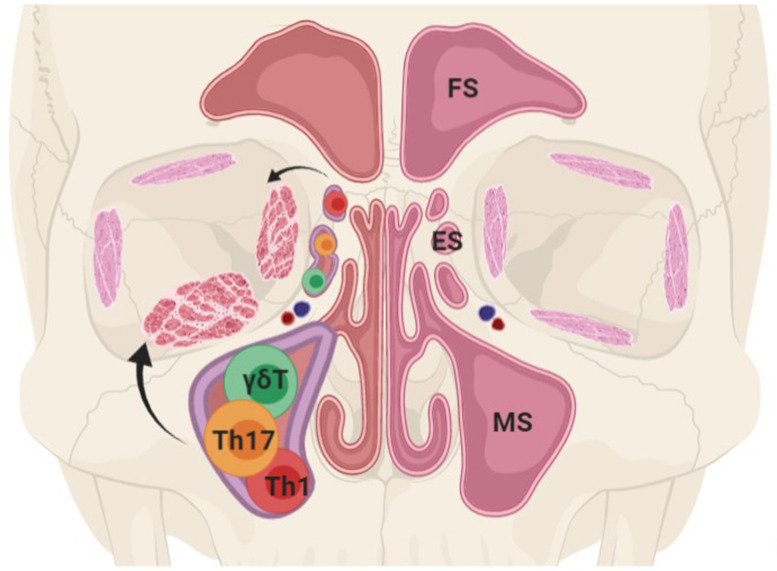
Theoretic model for the relationship between immunological abnormality in nasal sinus of Graves’ orbitopathy (GO) patients and orbital inflammation. FS, frontal sinus; ES, ethmoid sinus; MS, maxillary sinus (Declaration: this figure was designed by the first author Yi Lu with the help of drawing website “https://biorender.com”).

This research helps us build a preliminary understanding of the relationship between sinus disorder and GO. It could represent the local effects of sinus for the adjacent orbital inflammation. Inflammatory cells and cytokines may spread from the sinus to the orbit *via* natural dehiscence in adjacent bony walls or by hematogenous spread through common blood vessels (**Figure 5**). All in all, the immune abnormality of the nasal sinus mucosa in GO patients may not only be a clinical manifestation of Graves’ disease, but also be involved in the pathogenesis of GO.

## Data Availability Statement

The original contributions presented in the study are included in the article/supplementary material. Further inquiries can be directed to the corresponding author.

## Ethics Statement

The studies involving human participants were reviewed and approved by Ethic Committee of Shanghai Ninth People’s Hospital. The patients/participants provided their written informed consent to participate in this study.

## Author Contributions

Research conception and design: YLu, YW, SF, HZ. Experiment implementation: YLu, YW, JS, YLi. Analysis and interpretation of data: YLu, YH, SF. Drafting of manuscript: YLu, YW. Critical revision: SF, HZ. All authors contributed to the article and approved the submitted version.

## Funding

This work was supported by the National Natural Science Foundation of China (81930024, 82071003, 82000879, 81761168037, 81770974, 81800695, 81570883, 81600766, 31701046, 31600971, and 31500714), the Shanghai Sailing Program (18YF1412300), the Research Grant of the Shanghai Science and Technology Committee (20DZ2270800, 14JC1493103, 12419A9300, and 16411950600), the Shanghai Municipal Hospital Emerging Frontier Technology Joint Research Project (SHDC12012107), the Shanghai JiaoTong University School of Medicine Summit Plan, and the Shanghai JiaoTong University Medical and Engineering Cross Fund (YG2014MS03), the National Key R&D Program of China (2018YFC1106100, 2018YFC1106101), the Shanghai Municipal Hospital Emerging Frontier Technology Joint Research Project (16CR1004A), the Shanghai Municipal Education Commission—Gaofeng Clinical Medicine Grant Support (20152228), the Shanghai JiaoTong University Translational Medicine Crossed ResearchGrant (ZH2018ZDA12), and the Sample Database Project of Shanghai Ninth People’s Hospital (YBKB201901), the Joint Innovation Team for Young Physicians of Shanghai Ninth People's Hospital (QC202002).

## Conflict of Interest

The authors declare that the research was conducted in the absence of any commercial or financial relationships that could be construed as a potential conflict of interest.
